# HURRICANE KATRINA AND COVID-19: LASTING IMPACTS ON NEW ORLEANS’ AFRICAN AMERICAN WOMEN

**DOI:** 10.1093/geroni/igae098.0887

**Published:** 2024-12-31

**Authors:** Katie Cherry, Kori Balaam, Oliver Hardy-Smith, Piper Bordes, Kennedy Simon

**Affiliations:** Louisiana State University, Baton Rouge, Louisiana, United States; Louisiana State University, Baton Rouge, Louisiana, United States; Louisiana State University, Baton Rouge, Louisiana, United States; Louisiana State University, Baton Rouge, Louisiana, United States; Louisiana State University, Baton Rouge, Louisiana, United States

## Abstract

In 2005, two Category 5 hurricanes slammed into south Louisiana. Katrina brought catastrophic damage and substantial loss of life across the US Gulf Coast. Four weeks later, Rita crippled southwest Louisiana with tremendous destruction. In this study, we tested younger and older women who self-identified as African American / Black. All lived in the greater New Orleans metropolitan region at the time of the 2005 hurricanes. They completed a structured storm questionnaire and open-ended questions that tapped their Katrina experience and the more recent Covid-19 pandemic. Qualitative analyses on their narrative responses yielded three core themes: (1) Katrina Stressors: Family and Community Disruption, which captured catastrophic losses of homes and neighborhoods. Salient and frequent stressors included the disruption of social networks and pivotal life events (wedding plans, graduations). (2) Religiosity and Post-Traumatic Growth: Seeing Silver Linings, where coping through religious beliefs and practices (meditation, prayer, bible reading) helped participants navigate a new life journey. However, some questioned God following Katrina and one reflected that she did not join a new faith community after being separated from her home church in New Orleans, LA. (3) Katrina and COVID-19: Environmental Trauma on a Global Scale. The COVID-19 pandemic meant adjusting to a new normal with limited access to resources and businesses, reminiscent of post-Katrina life. A pressing responsibility to protect the safety and well-being of their family was also mentioned. Overall, these data permit new insight into individual and community-level vulnerabilities and strength-based resilience resources in the years after devastating environmental events.

